# How working memory relates to children’s reading comprehension: the importance of domain-specificity in storage and processing

**DOI:** 10.1007/s11145-016-9665-5

**Published:** 2016-06-24

**Authors:** Suzan Nouwens, Margriet A. Groen, Ludo Verhoeven

**Affiliations:** Behavioural Science Institute, Radboud University, Montessorilaan 3, P.O. Box 9104, 6500 HE Nijmegen, The Netherlands

**Keywords:** Working memory, Storage, Domain-specificity, Reading comprehension, Children

## Abstract

Working memory is considered a well-established predictor of individual variation in reading comprehension in children and adults. However, how storage and processing capacities of working memory in both the phonological and semantic domain relate to reading comprehension is still unclear. In the current study, we investigated the contribution of phonological and semantic storage, and phonological and semantic processing to reading comprehension in 123 Dutch children in fifth grade. We conducted regression and mediation analyses to find out to what extent variation in reading comprehension could be explained by storage and processing capacities in both the phonological and the semantic domain, while controlling for children’s decoding and vocabulary. The analyses included tasks that reflect storage only, and working memory tasks that assess processing in addition to storage. Regression analysis including only storage tasks as predictor measures, revealed semantic storage to be a better predictor of reading comprehension than phonological storage. Adding phonological and semantic working memory tasks as additional predictors to the model showed that semantic working memory explained individual variation in reading comprehension over and above all other memory measures. Additional mediation analysis made it clear that semantic storage contributed indirectly to reading comprehension via semantic working memory, indicating that semantic storage tapped by working memory, in addition to processing capacities, explains individual variation in reading comprehension. It can thus be concluded that semantic storage plays a more important role in children’s reading comprehension than previously thought.

## Introduction

Working memory—the ability to store information while simultaneously carrying out processing operations—is a well-established predictor of individual variation in reading comprehension performance in both adults (Daneman & Merikle, 1996) and children (Cain, Oakhill, & Bryant, 2004a). In the literature, it is debated whether individual differences in reading comprehension are best explained by processing or storage capacities of working memory. Various studies support the view that processing capacities tapped by working memory tasks in both the phonological and the semantic domain are important in explaining variance in reading comprehension (Daneman & Merikle, 1996). The role of storage has been investigated in the phonological domain but is less clear in the semantic domain since studies have typically used storage measures that tap into storage of phonological information rather than into semantic information (Haarmann, Davelaar, & Usher, 2003). Although some studies with adults (Haarmann et al., 2003) and children with difficulties in reading comprehension (Nation, Adams, Bowyer-Crane, & Snowling, 1999; Nation & Snowling, 1998) have suggested a link between reading comprehension and semantic storage, it is currently unknown if semantic storage contributes to reading comprehension in typically developing children. Moreover, it is by no means clear what the relative contribution is of phonological and semantic storage, on one hand, and phonological and semantic working memory, on the other hand, to children’s reading comprehension. Furthermore, it is unclear how semantic storage, semantic working memory and reading comprehension are related. Therefore, in the present study, children’s reading comprehension were related to their storage and processing capacity, in both the phonological and semantic domain.

Reading comprehension is the product of a complex integration of knowledge and skills such as decoding (Lyon, 1995; Torgesen, 2000), vocabulary (Verhoeven & van Leeuwe, 2008), and syntactic (Cutting & Scarborough, 2006; Oakhill & Cain, 2011) and semantic processing (Nation et al., 1999; Torgesen, 2000). In addition, reading comprehension depends on higher-level control functions (Cain, 2006; Christopher et al., 2012), among which working memory is the most well-established predictor in both adults (Daneman & Merikle, 1996) and children (Cain et al., 2004a, b). A commonly applied working memory model in the reading comprehension literature, is the model of Baddeley and Hitch (1974; see also Baddeley, 2000). According to the original model, working memory is composed of a central executive and two storage components, namely the visuospatial sketchpath and the phonological loop, encoding visuospatial and verbal information, respectively. More specifically, the phonological loop temporarily preserves verbatim representations of presented words and keeps this information active and accessible during the performance of complex cognitive tasks, which is controlled by the central executive. Various memory tasks have been designed based on Baddeley’s (2000) model, including tasks measuring the storage of information only, and working memory tasks reflecting the processing component of the central executive, in addition to storage of information. Working memory measures have a higher predictive value of reading comprehension performance than measures that assess storage only in adults (Daneman & Merikle, 1996), children (Cain, 2006) and children with reading comprehension difficulties (Carretti, Borella, Cornoldi, & De Beni, 2009). These results have been taken to suggest that it is the general processing capacities tapped by working memory tasks that are important in explaining variance in reading comprehension, rather than the storage component (Cain et al., 2004a, b; Daneman & Merikle, 1996).

Indeed, working memory tasks explain variance in reading comprehension regardless of whether they mainly involve non-verbal processing (recall visual patterns and/or spatial traces) or verbal processing (Carretti et al., 2009; Daneman & Merikle, 1996). There is, however, substantial evidence that the linguistic information tapped by working memory tasks is of primary importance with regard to explaining variance in reading comprehension (Daneman & Merikle, 1996). There is considerable variation in the kind of language processing involved among the different types of verbal working memory tasks, ranging from tasks that tap mainly into phonological processing (e.g., backward digit span tasks) to tasks that tap mainly into semantic processing (e.g., listening span tasks), and tasks that lie somewhere in between. During a backward digit span task, participants are asked to recall verbally presented digits in reverse order. Hence, the task requires storage and processing of verbatim information that contains a minimal amount of syntactic and semantic relations between items. During a listening span task, participants listen to a set of unrelated sentences and judge if sentences are semantically correct or incorrect. After the set of sentences has been presented, participants are asked to recall the sentence-final words (Daneman & Carpenter, 1980). In addition to verbatim encoding, the listening span task requires participants to integrate the presented items based on syntactic and semantic information. In other words, the listening span task relies on processes that serve language comprehension (Hulme et al., 1997; Knott, Patterson, & Hodges, 1997; Walker & Hulme, 1999). Semantic working memory tasks have been shown to be better predictors of reading comprehension than working memory tasks that mainly tap phonological processing (Cain et al., 2004a, b; Daneman & Merikle, 1996; Oakhill, Cain, & Bryant, 2003; Seigneuric, Ehrlich, Oakhill, & Yuill, 2000) and non-verbal working memory tasks (Shah & Miyake, 1996) in both typically developing children and adults. Similarly, children with difficulties in reading comprehension have shown deficits solely in verbal working memory, with the most profound deficits on tasks mainly tapping into semantic processing (Cain, 2006; Cain, Oakhill, & Lemmon, 2004b; Carretti et al., 2009; De Beni, Palladino, Pazzaglia, & Cornoldi, 1998; Nation et al., 1999). This has lead to the claim that not all variation in working memory can be explained by general processing capacity, but that linguistic information tapped by memory tasks, must play an important role as well (Daneman & Merikle, 1996).

In a similar way, the degree to which tasks that measure storage only rely on semantic rather than phonological aspects of stored representations may influence the extent to which performance on storage tasks explains variation in reading comprehension. Studies investigating the role of storage in reading comprehension have commonly used measures such as the forward digit span task, which requires immediate verbatim recall of a number of items (digits, letters or words) in exact serial order, thought to take place in Baddeley’s phonological loop (Baddeley, 2000). Correlations between performance on phonological storage tasks and reading comprehension in children were not significant (Leather & Henry, 1994; Swanson & Berninger, 1995; Yuill, Oakhill, & Parkin, 1989) or very low (Daneman & Carpenter, 1980; LaPointe & Engle, 1990; Turner & Engle, 1989). Additionally, children with difficulties in reading comprehension performed similarly to controls on these types of storage tasks (Nation et al., 1999; Oakhill, Yuill, & Parkin, 1986; Stothard & Hulme, 1992). To summarize, phonological storage has been found to be a poor indicator of reading comprehension performance.

There are, however, several indications that the ability to store semantic information may contribute to individual variation in reading comprehension. Children with difficulties in reading comprehension do not appear to benefit from the availability of long-term semantic representations to the same extent as controls: children with reading comprehension difficulties show a poorer performance on the recall of abstract and low frequency words compared to the control children, but perform similarly on the recall of concrete and high frequency words, suggesting that the deficiencies lie in the recall of semantic information (Nation et al., 1999; Nation & Snowling, 1998). Moreover, Haarmann et al. (2003) have shown that the conceptual span task designed to tap mainly into semantic storage explained unique variance in adult reading comprehension over and above a word span task. Based on these results it can be hypothesize that semantic, rather than phonological information tapped in storage tasks may explain variation in reading comprehension. Moreover, these results question the assumption that it is mainly the general processing component tapped by working memory tasks, rather than storage of the items involved, that is important in explaining variance in reading comprehension.

However, to our knowledge, research into the contribution of semantic storage and inherently, the relative contribution of phonological and semantic storage, on one hand, and phonological and semantic processing, on the other hand, to reading comprehension, has not yet been reported. Additionally, although Daneman and Carpenter’s listening span task (1980), is assumed to reflect simultaneous storage and processing of semantic information, the contribution of semantic storage to performance on the listening span task has not been explicitly investigated. Insight in this matter would be useful as the listening task is frequently used to assess working memory in the reading comprehension literature.

In the present study we aimed to examine the relation between phonological and semantic storage and processing capacities and reading comprehension in Dutch fifth grade children, after controlling for their vocabulary and word decoding. More specifically, we posed four research questions. The first question relates to the contribution of the phonological and semantic storage measures to reading comprehension:

1. Is semantic, but not phonological, storage a direct predictor of reading comprehension?

The other three questions speak to a model in which semantic and phonological working memory tasks, which assess processing in addition to storage, were added to the model:

2. Is processing, but not storage, a direct predictor of reading comprehension?
3. Is semantic, but not phonological, processing a direct predictor of reading comprehension?
4. If so, does semantic storage indirectly predict reading comprehension via semantic working memory?

## Method

### Participants

A total of 123 Dutch fifth grade children was recruited from four elementary schools in the Netherlands. Six children were excluded from the sample, including (1) four children who scored over 2.5 SDs below the group mean (*M* = 32.2, *SD* = 3.1) on our measure of non-verbal cognitive ability, which ranks below the 25th percentile of Dutch children (Raven, Raven, & Court, 2003), and (2) two children who failed to answer over 10 % of the questions on the reading comprehension test.

The final sample included 117 children, consisting of 62 boys (53 %) and 55 girls aged between 9 years and 9 months and 12 years and 1 month (*M* = 11.1 years, *SD* = .43). Children diagnosed with a developmental disorder were included to increase the statistical power of the results. The sample included 14 children with dyslexia, 12 children with ADHD, 2 children with Asperger Syndrome, and one child with comorbid disorders including ADHD, dyslexia and dyspraxia.^1^ The percentage of children that were non-native speakers of Dutch (<3 %) fell below the average minority representation (15 %) in Dutch elementary school (Tesser, Merens, & van Praag, 1999). Informed parental consent was obtained for all children.

### Materials

#### Reading comprehension

Reading comprehension was assessed using the standardized Dutch test “Diatekst” (H. I. Hacquebord, personal communication, school year 2011–2012). The test consisted of six texts with an average difficulty level suitable for grade five. The children were instructed to read the text before answering the questions. The test included 10–12 multiple-choice questions per text covering information that was either explicitly or implicitly stated in the text. The texts were available for reading during the entire test. On average, it took participants 30 min to finish the test. All participants finished within 60 min. Reading comprehension reflected the total number of correct answers (maximum = 67). The reliability analyses revealed a Cronbach’s alpha of .89 for this measure.

#### Non-verbal cognitive ability

The Raven’s Coloured Progressive Matrices (Raven et al., 2003) was used to assess non-verbal cognitive ability. The test comprised three sets (A, Ab, B) of 12 items. The items consisted of a visual pattern with a missing element. Participants were required to identify the missing element that could complete a pattern, choosing from six alternatives. The items were arranged in order of increasing difficulty. The number of items correct reflected non-verbal cognitive ability. The maximum possible score was 36.

Internal consistency is reported to be .76 for 11 year olds and the split-half reliability is reported to be .81 for 10 and 11 year olds (Cotton et al., 2005).

#### Decoding

A standardized Dutch test, the Klepel (Brus & Voeten, 1999), was used to assess decoding skills. Participants were instructed to read a list of pseudowords as fast and accurately as possible. The pseudowords on the list increased in difficulty. The total score reflected the number of pseudowords read correctly within 2 min. The maximum possible score was 116. The parallel-forms correlation for grade 5 is .92.

#### Vocabulary

Receptive vocabulary was assessed with the Peabody Picture Vocabulary Test-III-NL (Dunn & Dunn, 2005). Participants were presented with four pictures and were asked to select the picture that best reflected the verbally presented word. Words were presented in blocks of 12 items. The task ended when participants made nine errors or more within one block. The maximum possible score was 204. The internal consistency was reported .95 for 11 year olds.

#### Phonological storage

Phonological storage was assessed with the Forward Digit Span (Wechsler, 1992). Participants were required to recall a string of digits in the presented order. The test consisted of eight blocks, which each contained two trials. The number of digits to be recalled, increased over blocks, starting with two and ending with nine digits. The test ended when the participant incorrectly recalled both trials within a block. The number of correctly recalled trials reflected phonological storage. The maximum possible score was 16. The internal consistency reliability for this task was calculated as .78.

#### Semantic storage

Semantic storage was assessed with a Dutch translation of the conceptual span test designed by Haarmann et al. (2003). This conceptual span task consisted of 16 trials, each trial including a randomly ordered list of nine nouns that fitted in three different semantic categories (three nouns per category). The nine nouns were presented sequentially in small letters, with a rate of one word per second. Participants read the words silently from the computer screen. After the presentation of the nouns, one of the three category names was presented in capital letters. Participants were asked recall the three nouns (in any order) that fitted into the presented category. For instance, participants would see the following sequence of words: *lamp, pear, tiger, apple, grape, elephant, horse, fax, phone,* followed by the word *fruit?* In which case the correct answer would have been: pear, grape, apple. Compared to semantic working memory tasks, the involvement of processing is limited in the conceptual span task, as this task does not involve sentence processing and hence, the need for participants to integrate the presented items based on syntactic and semantic information. Moreover, unlike working memory tasks, the conceptual span task does not include dual-task requirements, which also limits the involvement of processing. The conceptual span task differs from the phonological storage measures, as the category-cued recall component of the conceptual span task is likely to engage activation of semantic storage. The contribution of phonological storage was minimized by using high-frequency words and by pre-exposing the participants to all 48 nouns and all six categories prior to the test. Participants were asked to read each word aloud and think about how the word fitted into the relevant category. This procedure was done twice in succession prior to the start of the experimental blocks. Concurrently, this procedure reduced the possibility of long-term memory intrusions (naming non-task related nouns). The materials and procedure were adapted from Haarmann et al. (2003) and were translated into Dutch. In order to prevent phonological and semantic overlap in consecutive words in the Dutch translations, two items were replaced with different target words belonging to the same category. Specifically, in trial six, ‘appel’ (apple) was replaced by ‘peer’ (pear) as it overlapped with ‘sinaasappel’ (orange) and in trial 16, ‘oog’ (eye) was replaced by ‘maag’ (stomach) as it overlapped with ‘elleboog’ (elbow). The score on the conceptual span was defined as the number of words recalled correctly across the 16 trials. The maximum possible score was 48. In Haarmann et al.’s (2003) studies with adults, the split-half reliability of the conceptual span test was .85 after Spearman–Brown correction for test length. In the current study, the split-half reliability was .52. An additional split-value reliability-analysis on data obtained from adults (N = 17) performing the Dutch translation of the conceptual span task yielded a Chronbach’s alpha of .64.

#### Phonological working memory

Phonological working memory was assessed with the Backward Digit Span (Wechsler, 1992). In the backward condition of the digit span test, participants were required to reproduce the presented digits in reverse order. The backward digit span consisted of seven blocks. The number of digits increased over blocks, starting with two, and ending with eight digits. The test ended when the participant incorrectly recalled both trials within a block. The number of correctly recalled trials reflected phonological working memory. The maximum possible score was 14. The internal consistency reliability for this test was calculated as .70.

#### Semantic working memory

Semantic working memory was assessed with the translation of Gaulin and Campbell’s (1994) Competing Language Processing Task.^2^ The task was designed specifically for children, by including shorter and simpler sentences than those used in Daneman and Carpenter’s (1980) listening span test. The task included sets of unrelated sentences that were presented orally to the participants. Participants were instructed to judge if sentences were semantically correct or incorrect. After a set of sentences was completed, participants were requested to recall the sentence-final words in any particular order. The test started off with two sets containing two sentences each, and was followed by two sets each containing three sentences, leading up to six sentences per set. The number of correct judgments (whether sentences were semantically correct or in correct) was registered. All participants scored over 90 % correct. The total number of correctly recalled words was taken as an indication of working memory. The maximum possible score was 42. The split-half reliability of the task (calculated by dividing the equal sized sets) was .67 after Spearman–Brown correction for test length.

### Procedure

Non-verbal cognitive ability and reading comprehension were administered in the classroom. The remaining tasks were administered individually and divided over two sessions. The order of the tasks was fixed and carefully arranged to prevent cognitive overload. All reported scores are raw scores.

### Data analyses

The analyses comprised correlations, hierarchal regression and mediation analyses. The regression analysis consisted of the following models: To ensure that the explained variances of the memory tasks were not due to individual differences in vocabulary or technical reading skill, vocabulary and decoding measures were entered in a first step (Model 0). Phonological and semantic storage tasks were entered in the second step (Model 1). Working memory measures were added in the last step (Model 2). At every step, all relevant variables were entered simultaneously. To investigate the relation between semantic storage, semantic working memory as assessed with the listening span task and reading comprehension, a mediation analysis was performed, using the bootstrapping procedure of Preacher and Hayes (2004).

## Results

Descriptive statistics and correlations are displayed in Tables 1 and 2, respectively. All variables met the requirements for normal distribution as skewness and kurtosis were >−2.0 and <2.0 (cf. George & Mallery, 2010). As can be seen in Table 2, decoding had a moderate correlation with reading comprehension, which is comparable to the results of other studies in which reading comprehension was investigated in transparent languages (e.g., Seigneuric & Ehrlich, 2005; Veenendaal, Groen, & Verhoeven, 2014). Vocabulary also showed a moderate correlation with reading comprehension, which is in line with other studies investigating the relation between receptive vocabulary, and reading comprehension in children of a similar age (Verhoeven & van Leeuwe, 2008).

**Table 1:** Descriptive statistics including mean scores and standard deviations (SD), minimum (Min.) and maximum (Max.) scores, skewness (Skew.) and kurtosis (Kurt.) values including standard errors (SE)

	Mean	SD	Min.	Max.	Skew.	SE	Kurt.	SE
Reading comprehension	50.79	9.83	25	66	−.84	.22	−.04	.44
Decoding	63.08	18.43	22	102	.10	.22	−.40	.45
Vocabulary	124.52	14.00	96	155	−.39	.22	−.25	.45
Memory tasks								
Phonological storage	7.58	1.33	4	11	.43	.22	.22	.44
Semantic storage	30.05	4.23	20	40	−.20	.22	−.48	.44
Phonological working memory	4.85	1.30	2	8	>.01	.22	.16	.44
Semantic working memory	26.79	3.60	17	38	.23	.22	.49	.44

**Table 2:** Bivariate two-tailed correlations among reading comprehension, decoding, vocabulary and the four memory tasks

	1.	2.	3.	4.	5.	6.	7.
1. Reading comprehension	–						
2. Decoding	.32**	–					
3. Vocabulary	.37**	.17	–				
4. Phonological storage	.16	.20*	.15	–			
5. Semantic storage	.22*	.11	.04	.21*	–		
6. Phonological working memory	.16	.27**	.22*	.55**	.29**	–	
7. Semantic working memory	.36**	.14	.14	.43**	.42**	.30**	–

* *p* < .05; ** *p* < .01

A hierarchical regression was performed to assess the contribution of performance on phonological and semantic storage tasks, and phonological and semantic working memory tasks to reading comprehension (see Table 3), after controlling for decoding and vocabulary. In the first step (Model 0), we investigated the relative contribution of decoding and vocabulary. Both explained unique variance in reading comprehension, F(2,113) = 14.20, *p* < .001, adjusted *R* ^2^ = .19. When the two storage measures were added in a second step (Model 1), only performance on the semantic storage task contributed significantly to reading comprehension performance, F(4,111) = 8.91, *p* < .001, adjusted *R* ^2^ = .22, in addition to decoding and vocabulary. The contribution of performance on the phonological storage memory task was not significant. Phonological and semantic working memory tasks were entered in a final step (Model 2). Out of the four memory tasks, only performance on the semantic working memory task contributed to reading comprehension performance in this model, F(9,109) = 7.65, *p* < .001, added *R* ^2^ = .26. Interestingly, the addition of the working memory tasks in the final step led to a noticeable change in the beta-value of the semantic storage task, resulting in a no longer significant contribution of this task to reading comprehension. These results suggest that semantic storage may contribute to semantic working memory, which in turn may contribute to reading comprehension. In other words, semantic storage may contribute to reading comprehension via semantic working memory. To explore the relation between semantic storage, semantic working memory and reading comprehension, a mediation analysis was performed, using the bootstrapping procedure of Preacher and Hayes (2004).

**Table 3:** Hierarchical regression analysis with reading comprehension as the dependent variable

Model		B	SD	*β*	Sig.
0	Decoding	.1390	.05	.26	.003
	Vocabulary	.2270	.06	.32	<.001
1	Decoding	.1230	.05	.23	.010
	Vocabulary	.2190	.06	.31	<.001
	Phonological short-term memory	.3920	.64	.05	.540
	Semantic short-term memory	.4580	.20	.20	.022
2	Decoding	.1230	.05	.23	.007
	Vocabulary	.2100	.06	.30	<.001
	Phonological short-term memory	−.2160	.75	−.03	.775
	Semantic short-term memory	.2470	.21	.11	.246
	Phonological working memory	−.2670	.76	−.04	.725
	Semantic working memory	.7390	.26	.27	.006

**Fig. 1:**
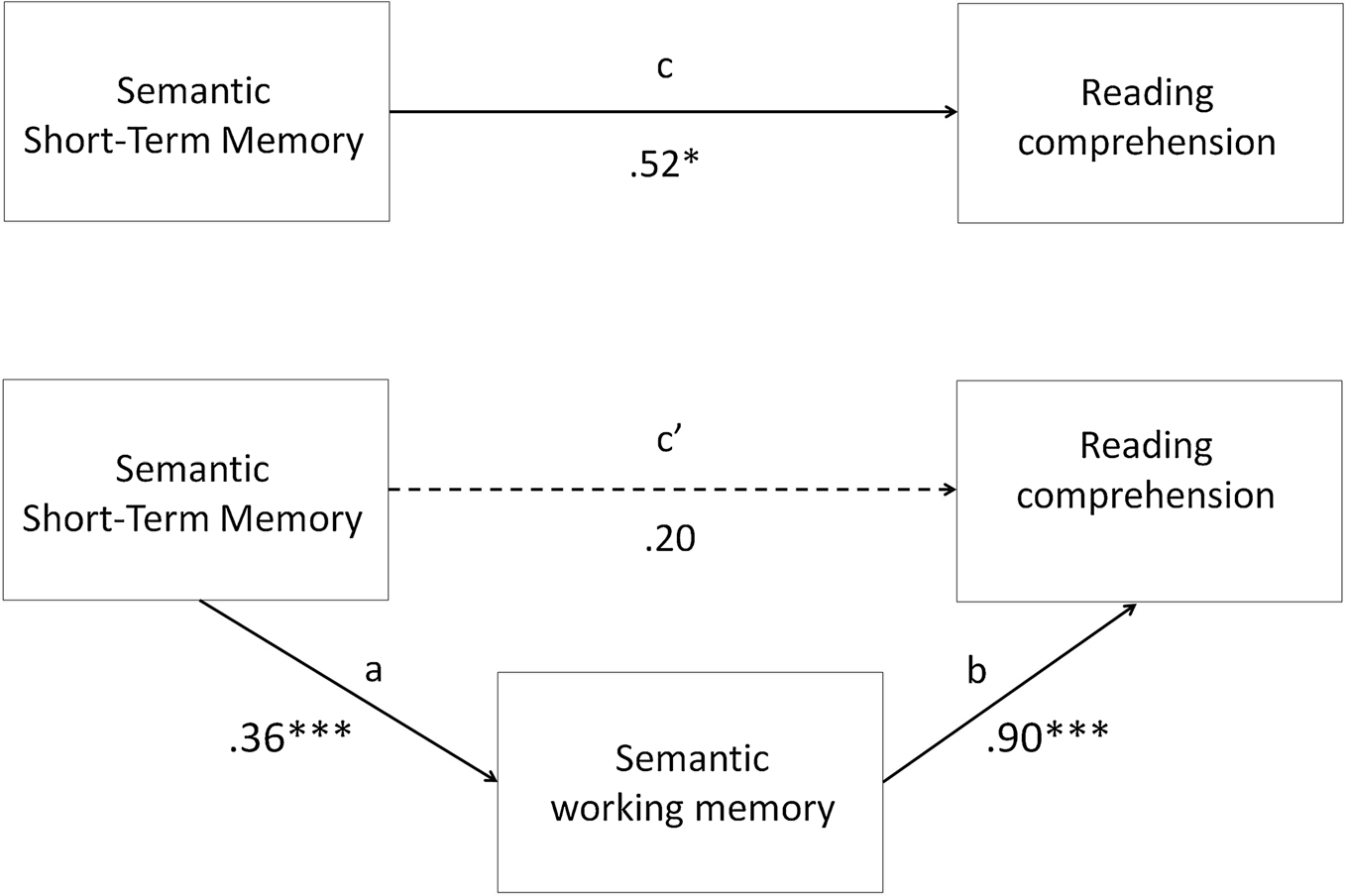
Mediation of the relation between semantic storage and reading comprehension by semantic working memory*. Note* *p < .05; **p < .01; ***p < .001. The values belonging to the *ab* path were: *β* = .32, *SD* = .21, *p* = .005

As depicted in Fig. 1, the direct relation between performance on the semantic storage task and reading comprehension was significant (*c* path; *β* = .52, *SD* = .21, *p* = .016), indicating that the level of semantic storage capacity predicted the level of reading comprehension. Additionally, performance on the semantic storage task contributed significantly to semantic working memory (*a* path; *β* = .36, *SD* = .07, *p* < .001), and working memory in turn contributed significantly to reading comprehension performance (*b* path; *β* = .90, *SD* = .26, *p* < .001). The indirect relation of performance on the semantic storage task to reading comprehension via working memory (*ab* path; *β* = .32, *SD* = .21, *p* = .005) to reading comprehension was also significant. However, when the whole model was taken into consideration, the initial significant relation between performance on the semantic storage task and reading comprehension (*c*′ path; *β* = .20, *SD* = .22, *p* = .379) was no longer significant. In other words, semantic storage only had a indirect contribution to reading comprehension via working memory. The whole model explained 12 % of reading comprehension and was significant, F(2,113) = 9.176, *p* < .001. Moreover, the bias-corrected 95 % confidence intervals for the indirect effect did not include zero, which confirms the significance of the findings.

## Discussion

The aim of the present study was to investigate the contribution of phonological and semantic storage, and phonological and semantic working memory to children’s reading comprehension, while accounting for decoding and vocabulary. By doing so, we examined the relative contribution of storage and processing capacities of working memory, while focusing on different aspects of linguistic information tapped by memory measures. We asked ourselves (1) whether semantic, but not phonological, storage is a direct predictor of reading comprehension, if measures assessing storage only are involved, (2) whether processing, but not storage, is a direct predictor of reading comprehension if phonological and semantic working memory measures are added to the analyses, (3) whether semantic, but not phonological processing is a direct predictor of reading comprehension, and (4) whether semantic storage indirectly predicts reading comprehension via semantic working memory.

The regression analysis with the two storage capacities as predictors revealed that semantic storage contributed to reading comprehension, while the contribution of phonological storage was not significant. These results are in line with previous studies that demonstrate no relation between phonological storage and reading comprehension in typically developing children (Leather & Henry, 1994; Oakhill et al., 1986; Yuill et al., 1989) and in children with reading comprehension difficulties (Nation et al., 1999; Oakhill et al., 1986; Stothard & Hulme, 1992). Additionally, these results fit well with results found in children with comprehension difficulties, who do not appear to benefit from the availability of long-term semantic representations in the same way as controls when asked to recall verbal stimuli (Nation et al., 1999). Moreover our results fit with Haarmann et al. (2003) who showed that semantic storage (also assessed with a conceptual span task) explained unique variance in reading comprehension beyond the measures of phonological storage in adults.

Addition of working memory measures to the regression analysis revealed that, similarly to the storage measures, the semantic working memory measure was a better predictor of reading comprehension than the phonological working memory measure, which is consistent with the results of previous studies in adults (Daneman & Merikle, 1996) children (Oakhill et al., 2003; Seigneuricet al., 2000) and children with reading comprehension difficulties (Cain et al., 2004a, b). These results suggest that linguistic information tapped by working memory measures influences the extent to which they explain variation in reading comprehension Additionally, the semantic working memory measure was a better predictor of reading comprehension than the storage measures, which is also in line with previous studies including adults (Daneman & Merikle, 1996), typically developing children (Cain, 2006) and children with reading comprehension difficulties (Carretti et al., 2009). These results support the view that general processing capacities tapped by working memory tasks are more important in explaining variance in reading comprehension than storage capacities (Daneman & Merikle, 1996; Cain et al., 2004a, b). Importantly, the mediation analysis revealed that semantic storage contributes to reading comprehension via semantic working memory. Hence, the current study shows that semantic storage capacity tapped by working memory tasks, in addition to general processing capacities, explained variance in reading comprehension, which has been proposed by a small number of previous studies (Haarmann, Just, & Carpenter, 1997; Nation & Snowling, 1998). It is interesting to note that our results are fully commensurate with behavioral studies in patients (Hanten & Martin, 2000; Martin & He, 2004) and neuro-imaging studies in healthy adults (Martin, 2015; Martin, Shelton, & Yaffee, 1994) that have proposed of a separate semantic storage component, in addition to the phonological loop of Baddeley’s model (2000).

At first glance, the results of the current study appear to be in contrast with the result found by Haarmann et al. (2003) who found a unique contribution of semantic storage to adults’ reading comprehension, even when working memory was included in the model. However, the subtle differences in type of working memory tasks and reading comprehension tasks used can explain the differences in findings. Haarmann et al. (2003) hypothesized that the involvement of semantic storage may depend on the type of reading comprehension task, as they did not find a unique contribution of semantic storage to all used reading comprehension tasks when working memory measures were included. They suggested that semantic storage becomes more important in reading comprehension when there is greater need for domain-specific linguistic skills.

The present study can be seen as a first step in uncovering the complex relations between phonological and semantic storage and phonological and semantic working memory, and reading comprehension. It should be noted that, in the current study, the reliability coefficients of the semantic memory tasks were relatively low, which may be caused by the small sample size of our study. Moreover, as we opted for the use of mostly standardized and often used memory tasks, the involvement of control processes may differ across them. Specifically, whereas the contribution of control processes is likely to be minimal in the phonological storage task (forward digit span), the semantic storage task (conceptual span) might involve some updating of information (see also Kane & Miyake, 2007). Together, this warrants caution in the interpretation of the results. The results therefore await replication in follow-up studies including multiple measures to reflect constructs that are either carefully matched and/or vary on the continuums of both phonological and semantic contributions as well on storage and processing.

In addition, it may be of interest to study the relation between semantic storage and reading comprehension in children with reading comprehension difficulties. It has been proposed that semantic storage aids in maintaining lexical-semantic item representations (Potter, 1993; Potter & Lombardi, 1990). Hence, a low semantic storage capacity may fail to aid in the integration of lexical-semantic item representations, which in turn may lead to reading comprehension problems (Haarmann et al., 1997; Martin, 2015). This possibility should be studied further in future work.

To conclude, the current study showed that semantic storage was a better predictor of individual variation in reading comprehension than phonological storage, indicating that the degree to which semantic information is tapped by storage tasks influences the extent to which these tasks explain variation in reading comprehension. Furthermore, it was found that the semantic working memory task explained individual variance in reading comprehension over and above all other memory measures. Importantly, the current study also showed that semantic storage contributed to reading comprehension via semantic working memory, indicating that both semantic storage and processing components tapped by working memory are important in explaining individual variation in children’s reading comprehension.

## Footnotes

1 Based on additional analyses, we concluded that the inclusion of children with diagnoses of developmental disorders did not influence the results. The ANOVA with group (dyslexia, ADHD, Asperger Syndrome and typically developing) as between-subjects factor demonstrated that children with a diagnosis did not differ in their performance on the memory tasks from typically developing children. However, group differences were found for reading comprehension, F(3,114) = 5.84, *p* = .001, where both children with dyslexia and children with ADHD scored lower than the other children. Group differences were also found for decoding, F(3,113) = 10.53, *p* < .001, due to the dyslexia group scoring lower on decoding than typically developing children (*p* < .001). Moreover, there were no interactions with the independent variables and dyslexia or ADHD on the regression analysis with reading comprehension as the dependent variable (the interaction variables all had a p value that exceeded .10). Additionally, the data was checked for influential cases. Based on Cook’s distance, leverage values and Mahalanobis values it could be concluded that there were no influential cases (Tabachnick & Fidell, 2007).

2 The materials and procedure were adapted from Gaulin and Campbell (1994) and were translated into Dutch. For the current study we made two minor adaptions. “Pumpkins are purple” was translated into “Mandarijnen zijn paars” (Mandarins are purple) and “Hotdogs can bark” into “Koeien kunnen blaffen” (“Cows can bark”) as “Pumpkins” and “hotdogs” are not high frequency words in Dutch.
